# Associations of stress, anxiety, and partner satisfaction with maternal-fetal attachment in women pregnant during the COVID-19 pandemic: an online study

**DOI:** 10.1186/s12884-023-05804-1

**Published:** 2023-06-30

**Authors:** Nora K. Schaal, Pearl La Marca-Ghaemmaghami, Sarah Märthesheimer, Philip Hepp, Heidi Preis, Brittain Mahaffey, Marci Lobel, Rita Amiel Castro

**Affiliations:** 1 Institut für Experimentelle Psychologie, Heinrich-Heine-Universität Düsseldorf, Universitätsstraße 1, 40225 Düsseldorf, Germany; 2 Psychology Counselling and Research Institute for Sexuality, Marriage, and Family, International Academy for Human Sciences and Culture, Walenstadt, Switzerland; 3 Clinic for Gynecology and Obstetrics, KJF Klinik Josefinum gGmbH, Augsburg, Germany; 4 Clinic for Gynecology and Obstetrics, HELIOS University Hospital Wuppertal, University Witten/Herdecke, Wuppertal, Germany; 5 Department of Psychology, Stony Brook University, Stony Brook, NY USA; 6 Department of Pediatrics, Renaissance School of Medicine, Stony Brook University, Stony Brook, NY USA; 7 Department of Psychiatry and Behavioral Health, Renaissance School of Medicine, Stony Brook University, Stony Brook, NY USA; 8 Department of Clinical Psychology and Psychotherapy, University of Zurich, Zurich, Switzerland

**Keywords:** COVID-19 pandemic, Stress, Anxiety, Partnership satisfaction, Prenatal attachment

## Abstract

**Background:**

The COVID-19 pandemic has led to exceptional stress in pregnant women. The aim of the present study was to investigate associations of maternal stress (pandemic-related and -unrelated), anxiety, and relationship satisfaction experienced during the COVID-19 pandemic with prenatal mother-infant attachment.

**Methods:**

An online study was conducted evaluating pandemic-related stress, pregnancy-specific stress (unrelated to the pandemic), anxiety, partnership satisfaction, and maternal-fetal attachment in German-speaking women during the second COVID-19 lockdown between January and March 2021. In total, 431 pregnant women (349 lived in Germany and 82 in Switzerland) filled in the questionnaires and gave information on demographic and pregnancy-related variables (i.e. age, gestational age, parity). Bivariate correlations were calculated in order to investigate associations between the different variables and additionally, a hierarchical regression model was conducted in order to evaluate the influence of the independent variables on prenatal attachment.

**Results:**

The hierarchical regression analysis revealed that after controlling for age, gestational age, and parity higher pandemic-related stress, namely stress associated with feeling unprepared for birth, higher partnership satisfaction as well as higher positive appraisal (considered as a way of coping with pandemic-related stress) was associated with stronger maternal-fetal attachment, whereas associations of anxiety and other forms of stress were non-significant.

**Conclusions:**

The study highlights interesting associations between maternal pandemic-related preparedness stress and positive appraisal of the pregnancy as well as partnership satisfaction and prenatal attachment in women pregnant during the COVID-19 pandemic.

## Introduction

The COVID-19 pandemic has had a great impact on the world population, affecting many areas of life since 2020 [1]. Pregnant women in particular reported elevated stress and anxiety due to COVID-19 restrictions, uncertainty about the circumstances under which they would give birth, and how COVID-19 might affect their own and their baby’s health [2–4]. Although some studies have shown that direct transmission of the virus to the fetus is rare [5, 6], others have found adverse effects on birth outcomes in infected pregnant women. These include preterm birth [7, 8], admissions to the NICU [9], and more severe COVID-19 symptoms in pregnant women including higher risks of maternal admission to intensive care and risk for mechanical ventilation [10, 11]. Anxiety and stress in pregnant women are affected not only by these medical risks, but also by social consequences of the pandemic, such as social distancing, isolation, and reduced social support [12–15]. However, whether elevated COVID-19-related stress affects other important aspects of pregnancy such as prenatal attachment and partner relationships has not received much attention.

Lower maternal-fetal attachment in the prenatal period has been linked to lower mother-infant attachment postnatally [16–18]. Poorer attachment can have far-reaching consequences for mother and child, including higher maternal anxiety and lower emotional regulation abilities in the child [19, 20]. Prior studies have shown that higher prenatal maternal stress, anxiety, and diminished mental health during pregnancy can adversely influence mother-infant attachment [18, 21], consequently impairing children’s later development [20]. Recent studies also suggest that prenatal attachment has been lower in women pregnant during the COVID-19 pandemic compared to women pregnant before the pandemic [22, 23]. A study investigating the impact of mental health and other psychological factors on maternal-fetal attachment during the first wave of the COVID-19 pandemic reported that depressive symptomatology was associated with lower attachment, while a psychiatric history and distress tolerance were unrelated to attachment [24]. However, the study also showed that COVID-19-related health worries, anxiety, and resilience were protective factors, associated with stronger attachment. This mixed pattern of findings underscores the need for additional research in pregnancy to clarify links between COVID-19-related stress and emotional states with prenatal maternal attachment.

In the current study, we also examined relationship satisfaction as a contributor to prenatal attachment as studies have shown that together with marital status, partnership satisfaction positively influences prenatal and postnatal mother-infant attachment [25, 26]. Matthies and colleagues [26] found that the relationship between prenatal maternal-fetal attachment and postpartum anxiety was mediated by relationship satisfaction. This highlights that attachment to one’s unborn child and to one’s partner together may influence postpartum adjustment.

The present study evaluated levels of pandemic-related and pregnancy-specific (pandemic-unrelated) stress, anxiety, relationship satisfaction, and prenatal mother-infant attachment in German-speaking pregnant women in Germany and Switzerland during the second lockdown (January-March 2021) of the COVID-19 pandemic. The aim of the study was to investigate associations of maternal stress, anxiety, and relationship satisfaction experienced during the COVID-19 pandemic with prenatal mother-infant attachment. We hypothesized that higher stress and anxiety would be associated with lower attachment and with less relationship satisfaction, and that higher relationship satisfaction would predict greater attachment.

## Methods

The present study is part of an ongoing project, the International COVID-19 Pregnancy Experiences (I-COPE) Study, which was initiated at the beginning of the COVID-19 pandemic in March 2020. The I-COPE Study investigates the experiences of pregnant and postpartum women with an emphasis on the extent and impact of COVID-19-related stress in the peripartum period [2, 27].

### Participants

Participants for the current study were recruited during the second lockdown of the COVID-19 pandemic in Germany and Switzerland between January and March 2021. In order to participate, women needed to currently be pregnant (all gestational ages eligible), over 18 years of age, and able to understand the German language sufficiently to complete the online study questionnaire. The main components of the survey (i.e. demographics, pandemic-related and pregnancy-specific stress, general anxiety) was developed by colleagues from the US [2] and translated to German by our team [27]. For the present study, the survey was expanded by including questionnaires evaluating maternal-fetal attachment and relationship satisfaction. Eligible women provided written informed consent before participating. The data were stored according to General Data Protection Regulation in Germany and Switzerland.

### Materials

The validated version of the *Maternal-Fetal Attachment Scale (MFAS)* was used to assess prenatal attachment [28]. The original version of the MFAS comprises 24 items [29]. However, as recommended by Sjögren et al. [30], we excluded 7 questions that refer to pregnant women’s reactions to the perception of fetal movements (e.g., “I enjoy watching my tummy jiggle as the baby kicks inside”) which can only be answered by women in later pregnancy. The 17 item version is thus more suitable when investigating women of all gestational ages [30], as was the case in the study at hand. Items (e.g., *“I talk to my unborn child”*) were rated using a 5-point Likert scale ranging from 1 = Never to 5 = Very often. A total score was calculated with higher scores indicating stronger attachment. Cronbach’s alpha for the MFAS in the current study was 0.783.

The *Pandemic-Related Pregnancy Stress Scale (PREPS)* is a 15-item instrument that assesses pregnancy-related stress originating from the COVID-19 pandemic. The PREPS was developed by Preis and colleagues [2, 31] at the beginning of the COVID-19 pandemic in the US. A German version was also published [27]. Participants rated each item on a 5-point Likert scale ranging from 1 (Very little) to 5 (Very much). Exploratory and confirmatory factor analyses of the instrument reveal that it includes three factors: (1) PREPS-Preparedness (7 items), which reflects stress regarding birth preparation and the early postpartum phase (e.g., *“I am worried that the pandemic could ruin my birth plans”*); (2) PREPS-Infection (5 items), which assesses stress about becoming infected with COVID-19 (e.g., *“I am worried that I might get COVID-19 when I go to the hospital to deliver”*), and (3) PREPS-Positive Appraisal (3 items), which encompasses favorable aspects of being pregnant during the pandemic (e.g., *“I feel that being pregnant is giving me strength during the pandemic”*). Confirmatory factor analyses from the German validation study of the PREPS showed acceptable fit [27, 32]. In the current study, Cronbach’s alphas for the three subscales were 0.719 (Positive Appraisal), 0.804 (Preparedness) and 0.838 (Infection)].

Pregnancy-specific stress unrelated to the pandemic was assessed with the German version of the *Revised Prenatal Distress Questionnaire (NuPDQ)* [33]. This instrument consists of 17 items which are rated on a 3-point Likert scale (0 = Not at all to 2 = Very much). Women indicate how “bothered, upset or worried” they feel about different aspects of their pregnancy and the upcoming birth (e.g., “whether the baby might come too early” or “pain during labor and delivery”). Higher scores indicate greater stress. The NuPDQ has strong psychometric properties [34] and exhibited good internal consistency in this study (Cronbach’s alpha = 0.755).

Relationship quality was evaluated with the short version of the *Relationship Satisfaction Questionnaire* (*Partnerschaftsfragebogen;* PFB) [35] which has nine items (e.g., *“He/she tells me that he/she cares about me”*) scored on a 4-point scale ranging from 0 = Never to 3 = Very often. A total score is calculated with higher scores indicating greater relationship satisfaction. The PFB also includes a separate item evaluating overall happiness with the relationship, scored on a seven-point scale from 0 = Very unhappy to 6 = Very happy. This item was not used in the present study. Cronbach’s alpha for the PFB was 0.836.

General anxiety was measured with the German version of the Generalized Anxiety Disorder-7 (GAD; [36, 37]). This instrument comprises 7 items assessing recent symptoms of general anxiety. Respondents rate the items on a 4-point Likert scale ranging from 0 = Not at all to 3 = Nearly every day. Higher scores indicate higher anxiety. For the present study, Cronbach’s alpha was 0.864.

### Procedure

The online survey was conducted via the platform Sosci-Survey (www.soscisurvey.de) in Germany and Unipark (www.unipark.de) in Switzerland. Participants were recruited via social media (e.g., Facebook posts, Facebook groups) and flyer distribution through different organizations for pregnant women (e.g., *Motherhood*). After reviewing study information and providing informed consent, participants completed the study questionnaire, which took approximately 15 min. In Germany, participants had the chance to enter a prize drawing to win one of ten 10 Euro Amazon vouchers, whereas in Switzerland, each participant was sent a small gift if they indicated they would want to receive one.

### Statistical analysis

Statistical analyses were performed using the IBM Statistical Package for the Social Sciences (SPSS Version 26 for Windows). Bivariate spearman correlations were first calculated among MFAS, PREPS, PFB, NuPDQ, and GAD scores. Following this, a linear hierarchical regression model was constructed to examine independent associations of study variables with prenatal attachment. In Block 1 of the model, we included demographic and pregnancy-related factors which have been shown to influence prenatal attachment (maternal age, gestational age, and parity); Block 2 added the psychological factors that were significantly correlated with MFAS scores.

Analyses were adjusted for the following *covariates*: maternal age (continuous variable), gestational age (continuous variable), and parity (nullipara vs. multipara) as these have been shown to be important predictors of prenatal attachment [38, 39].

We checked the variables for normality. As most of the variables were not normally distributed, non-parametric spearman correlations were applied. Regarding the linear hierarchical regression model, we checked after running the analysis whether the residuals are normally distributed using histograms, P&P Plots as well as the normality test, which confirmed that the residuals were normally distributed.

## Results

### Demographics

For the present study, 431 pregnant women participated, including 349 who lived in Germany and 82 in Switzerland. Their mean age was 32 years (range: 21–44, SD = 3.8) and their mean gestational age was 26 weeks (range: 5–42, SD = 9.3). Approximately half (n = 224; 52%) were expecting their first child. Nearly all participants (n = 427; 99.3%) were married or living with a partner as if married.

### Bivariate analysis

Table 1 gives an overview of the descriptive measures of the variables of interest.

**Table 1:** Overview of variables with descriptive measures

	Mean (SD)	Median (IQR)
MFAS	46.91 (9.29)	48 (42–54)
PREPS-Prep	2.85 (0.93)	2.86 (2.14–3.57)
PREPS-Inf	2.84 (1.02)	2.80 (2.00-3.60)
PREPS-Pos	2.44 (1.02)	2.33 (1.67–3.33)
PFB	16.54 (3.77)	17.00 (14.00–20.00)
Nu-PDQ	0.69 (0.29)	0.65 (0.47–0.88)
GAD	6.27 (4.30)	5.00 (3.00–9.00)

As shown in Table 2, bivariate Spearman correlations revealed that maternal-fetal attachment (MFAS) was positively correlated with pandemic-related preparation stress (PREPS-Prep), pandemic-related infection stress (PREPS-Inf), the positive appraisal dimension of the pandemic-related stress questionnaire (PREPS-Pos), and with relationship satisfaction (PFB), whereas no significant correlation of attachment was found with pregnancy-specific stress unrelated to the pandemic (NuPDQ) or general anxiety (GAD). Therefore, NuPDQ and GAD scores were not included in the following regression analysis examining independent predictors of attachment. Correlations of relationship satisfaction (PFB) with pandemic-related preparation stress and infection stress (PREPS-Prep and PREPS-Inf) were non-significant; however, relationship satisfaction was correlated with positive appraisal (PREPS-Pos), and inversely with anxiety (GAD).

Additional analyses revealed significant positive correlations of attachment with age (r = .152; p < .01) and gestational age (r = .435, p < .001) and a significant difference on the MFAS by parity (nullipara vs. multipara), U (431) = 17845.5, z = -4.14, p < .001. Nulliparous women reported higher prenatal attachment (Mdn = 50) than did multiparas (Mdn = 45).

**Table 2:** Spearman correlations among study variables

	1. MFAS	2. PRESP-Prep		3. PREPS-Inf	4. PREPS-Pos	5. PFB	6. NuPDQ	7. GAD	8. Parity
1. MFAS	/		0.204**	0.118*	0.231**	0.321**	0.046	0.079	− 0.119**
2. PREPS-Prep			/	0.603**	− 0.047	0.021	0.590**	0.473**	− 0.098*
3. PREPS-Inf				/	− 0.031	− 0.034	0.494**	0.391**	− 0.041
4. PREPS-Pos					/	0.116*	− 0.028	− 0.142**	− 0.220**
5. PFB						/	− 0.052	− 0.143**	− 0.298**
6. NuPDQ							/	0.416**	− 0.244**
7. GAD								/	0.156**
8. Parity									

** p < .001; * p < .05

### Regression analysis

A hierarchical regression model was constructed predicting the dependent variable MFAS, adjusting for maternal age, gestational age, and parity in Block 1 and then adding PREPS-Prep, PREPS-Inf, PREPS-Pos, and PFB scores as predictors in Block 2. As shown in Table 3, Model 1 was significant, F (3, 417) = 46.61, p < .001. All three variables (maternal age, gestational age, and parity) contributed significantly and together accounted for 25.1% of the variance in MFAS. After adding the Block 2 variables, the explained variance in MFAS increased to 37.5%. Model 2 was also significant, F (7, 413) = 35.44, p < .001. In Model 2, gestational age, PREPS-Prep, PREPS-Pos, and PFB were significant predictors of MFAS.

**Table 3:** Hierarchical regression analysis predicting MFAS

Variable	β	t	P	R	R^2^
**Model 1**				0.501	0.251
Age	− 0.103	-2.311	0.021		
Gestational age	0.452	10.640	< 0.001		
Parity	− 0.145	-3.254	0.001		
**Model 2**				0.613	0.375
Age	− 0.078	-1.874	0.062		
Gestational age	0.442	11.224	< 0.001		
Parity	− 0.012	− 0.266	0.791		
PREPS-Prep	0.143	2.847	0.005		
PREPS-Inf	0.067	1.367	0.173		
PREPS-Pos	0.162	4.025	< 0.001		
PFB	0.276	6.691	< 0.001		

As it was surprising that parity is highly significant in Model 1 and turns out non-significant in Model 2, we then ran the regression analysis again and excluded the variables which were significantly correlated with parity (see Table 2) one by one starting with the variable with the strongest correlation (i.e. PFB first, then PREPS-Prep and PREPS-Pos).

When excluding PFB the variable parity remains significant in Model 2 (see Table 4), whereas excluding PRESP-Prep or PREPS-Pos did not change the overall pattern.

**Table 4:** Model 2 of the hierarchical regression analysis predicting MFAS excluding PFB

Variable	β	t	P	R	R^2^
**Model 2**				0.557	0.310
Age	− 0.089	-2.063	0.040		
Gestational age	0.435	10.626	< 0.001		
Parity	− 0.100	-2.309	0.021		
PREPS-Prep	0.147	2.812	0.005		
PREPS-Inf	0.047	0.916	0.360		
PREPS-Pos	0.175	4.195	< 0.001		

## Discussion

The primary aim of the present study was to evaluate associations of pregnancy stress related and unrelated to the pandemic, anxiety, and relationship satisfaction with maternal-fetal attachment in women pregnant during the second wave of the COVID-19 pandemic. We were also interested in determining whether stress and anxiety were associated with lower relationship satisfaction. Results offered mixed support for our hypotheses. Unexpectedly, after controlling for age, parity and gestational age, one aspect of pandemic-related stress, namely stress associated with feeling unprepared for birth and the postpartum, was an independent predictor of stronger, not weaker, attachment. Consistent with hypotheses, relationship satisfaction was independently predictive of attachment, as was positive appraisal, which is considered as a way of coping with pandemic-related stress [40]. Interestingly, the factor parity turned out non-significant when including pandemic-related stress, positive appraisal and partner satisfaction to the model predicting attachment. However, when excluding partner satisfaction from the regression model, as partner satisfaction was highly correlated with parity, parity significantly predicts prenatal attachment again. Neither anxiety nor other forms of pregnancy stress predicted attachment. Additionally, positive appraisal and lower anxiety were associated with higher relationship satisfaction, but stress was not associated with relationship satisfaction. These interesting results highlight a complex interplay between pandemic related stress, anxiety, partnership satisfaction and prenatal attachment, however, as we unfortunately did not evaluate depressive symptoms in the present cohort results need to be interpreted with caution as prior research has shown that this is an important influencing factor for prenatal attachment [41].

The positive association between relationship satisfaction and maternal-fetal attachment corroborates prior research [25, 26]. However, as the present study investigated this association in women pregnant during the COVID-19 pandemic, our findings extend previous research by showing that relationship satisfaction is positively correlated with prenatal attachment even in circumstances associated with heightened stress. Similarly, the finding that more advanced gestational age was linked to stronger maternal-fetal attachment fits well with prior evidence [24, 38]. Attachment increases as pregnancy advances and women become more aware of their baby’s movements and size. Essentially, the pregnancy becomes more tangible.

Earlier evidence has shown that perinatal stress is associated with lower maternal-fetal and maternal-infant attachment [18, 21, 42] and recent studies indicate that women pregnant during the COVID-19 pandemic have experienced lower attachment compared with pregnant women studied prior to the pandemic [22, 23]. Therefore, we expected that stress, and especially pandemic-related stress, would be inversely associated with attachment. Although contrary to our hypothesis, the finding that pandemic stress due to lack of preparation for birth and the postpartum predicted higher attachment is in accordance with a recent study by Koire and colleagues [24], who conducted an online survey during the first wave of the COVID-19 pandemic in the United States. The authors evaluated maternal-fetal attachment in pregnant women and found that higher levels of worry regarding how COVID-19 might affect their health was associated with stronger attachment. Similarly, a study by Craig and colleagues [41] evaluating psychological distress, prenatal attachment, and COVID-19 risk perception in pregnant women in Italy in March 2020, also revealed a positive correlation between COVID-19 risk perception and prenatal attachment. Moreover, Craig et al. pointed out that the relationship between anxiety and prenatal attachment was stronger among women with high COVID-19 risk perception [41]. The PREPS-Preparedness scale used in the present study and which predicted higher attachment, measures stress associated with impending birth. This greater worry about one’s preparedness for childbirth and becoming a mother may reflect increased recognition of the stakes that are involved in possible pandemic impacts, resulting in stronger connection to one’s child and enhanced commitment to the role of becoming a mother [24]. Stress associated with fears of infection and stress unrelated to the pandemic may not have precipitated such increased connection with the baby. Results of this study therefore highlight the value of differentiating types of prenatal maternal stress in order to distinguish their associations with fetal and infant attachment and other outcomes.

Associations among the positive appraisal dimension of the PREPS, relationship satisfaction, and maternal-fetal attachment suggest that women who cope with pandemic stressors by maintaining a positive orientation toward pregnancy also experience greater connection to their child and to motherhood, and both positive coping and stronger attachment are likely undergirded by greater support from one’s partner. A supportive relationship with one’s partner may have been especially vital during the height of the pandemic when lockdowns and other restrictions created isolation from family, friends, and other sources of social support. In this context, it would be interesting to investigate how other problem- and emotion-focused coping mechanisms that have been shown to be successful when experiencing challenging situations [43], including in pregnancy [44], might also help pregnant women to manage COVID-19 pandemic stressors, and be facilitated by social support from one’s partner or others.

The influence of parity on prenatal attachment is interesting and complex. In Model 1 of the regression analysis when only including basic characteristics, parity is a highly significant factor. As expected, women receiving their first child report higher prenatal attachment scores than mothers who are not nullipara. This is in accordance with previous findings, however, the meta analysis of Yacheski and colleagues point out that the effect size of parity on prenatal attachment is small [39]. It can be hypothesized that other factors such as partnership satisfaction may cofound the relationship as the presented results highlight. Partnership satisfaction was moderately correlated with parity showing that partnership satisfaction was higher in nullipara and when including partnership satisfaction in the original regression model, parity does not significantly predict prenatal attachment anymore. In this respect, it is possible that an important factor in order to develop a strong attachment to the unborn child is that pregnant women have a stable environment, which includes high partnership satisfaction but also factors such as social and family support [45, 46]. And if this is present, a woman can build up a good attachment even when she already has one or more children which need her attention and care.

The present study had a variety of strengths, including its execution during a global public health crisis occurring at a unique time in modern history, its use of well-validated instruments, and its focus on understudied topics. Nevertheless, the study’s reliance on a convenience sample recruited via social media, and its exclusive use of self-report instruments may limit its generalizability. Furthermore, the study did not comprehensively evaluate other predictors of maternal attachment. For example, we unfortunately did not assess depressive symptoms, which have been shown to be linked to impaired prenatal and postnatal attachment [41, 47], were elevated in women pregnant during the COVID-19 pandemic [4, 48, 49], and shown in at least one prior study to be linked to lower maternal-fetal attachment during the pandemic [24]. This is a limitation to the study, as depressive symptoms play an important role for prenatal attachment and therefore our results need to be considered keeping this limitation in mind. Presumably our model would have benefitted from including depressive symptoms and the explained variance of the prenatal bonding scores would have increased. However, we believe that the influences of stress and partnership satisfaction on prenatal bonding, which we show in the present study, would persist even when depressive symptoms are considered. This assumption should be tested in a follow-up study.

The current study also presumed directions of association among variables measured at a single timepoint. It would be valuable to replicate these findings in a longitudinal study, and to examine how these variables change across the perinatal period and influence infant development postpartum. Pre-pandemic studies have shown that prenatal attachment is closely linked to postnatal attachment [17, 18] and postnatal attachment intensity and quality is associated with important developmental factors, such as emotional states (e.g., anxiety), behaviors, and social competencies in childhood [20, 50, 51]. Some research has already shown associations between pandemic-related stress and birth outcomes, including lower birth weight and younger gestational age at birth [52, 53]. Studies of more far-reaching effects of pandemic pregnancy on child development are now imperative.

The results of the present study also highlight some implications for pregnant women. They suggest that maternal stress does not need to have negative impacts on mother-infant attachment, but rather, that pandemic-related stress can even be beneficial as is can increase prenatal attachment when in is recognized and seen as a chance to cherish the pregnancy more. The results also highlight the importance of partnership satisfaction in order to build a strong bond to the unborn baby, as it can be assumed that if the women feel satisfied with their life circumstances (and partnership quality is an important factor here), they can more easily build up an attachment to the unborn child.

## Conclusions

The study revealed that higher maternal pandemic-related preparedness stress and positive appraisal of the pregnancy as well as higher partnership satisfaction are associated with stronger prenatal attachment in women pregnant during the COVID-19 pandemic. Furthermore, the results highlight associations between anxiety and positive appraisal and partnership satisfaction. Taken together, the study revealed interesting relationships between maternal stress, partnership satisfaction and maternal-fetal attachment. It would be desirable if future studies include depressive symptoms as an important prenatal psychological factor as well as to then build on these results and investigate how these associations develop postpartum and influence developmental factors of the child.

## Data Availability

The dataset used and analysed in the current study is available from the corresponding author on reasonable request.

## Abbreviations

MFAS: Maternal-Fetal Attachment Scale
PREPS: Pandemic-Related Pregnancy Stress Scale
PREPS-Prep: Pandemic-Related Pregnancy Stress Scale – Preparedness Stress
PREPS-Inf: Pandemic-Related Pregnancy Stress Scale – Infection Stress
PREPS-Pos: Pandemic-Related Pregnancy Stress Scale – Positive Appraisal
NuPDQ: Revised Prenatal Distress Questionnaire
PFB: Partnerschaftsfragebogen/Relationship Satisfaction Questionnaire
GAD: Generalized Anxiety Disorder-7
